# Effect of Inhaled Colistin on the Treatment of Ventilator-Associated Pneumonia due to Multi-drug Resistant Acinetobacter

**Published:** 2019-01

**Authors:** Omid Moradi Moghaddam, Mohammad Niakan Lahiji, Mahshid Talebi-Taher, Behnam Mahmoodiyeh

**Affiliations:** 1 Trauma and Injury Research Center, Department of Critical Care, Iran University of Medical Sciences, Tehran, Iran; 2 Department of Infectious Disease, School of Medicine, Iran University of Medical Sciences, Tehran, Iran; 3 Department of Anesthesiology, School of Medicine, Arak University of Medical Sciences, Arak, Iran.

**Keywords:** Pulmonary infection, ICU, Inhaled colistin

## Abstract

**Background:**

Ventilator-Associated Pneumonia (VAP) is a hospital pneumonia that is considered in patients on mechanical ventilation for at least 48 hours with symptoms of new lower respiratory tract infections being reported in them. The present study reviews the effect of adding inhaled colistin in the treatment of ventilator-induced pulmonary infections in Intensive Care Unit (ICU) patients.

**Materials and Methods:**

In this single blind clinical trial, patients admitted to the ICU with diagnosis of pulmonary infections caused by ventilator were investigated. In the treatment group, patients received 150 mg of colistin plus 1,000,000 units inhaled colistin every eight hours and in the control group only 300 mg of colistin every eight hours intravenously was given. Patients were followed up in terms of clinical findings for seven days after the initial diagnosis of infection.

**Results:**

The results of this study showed that administration of inhaled colistin in patients admitted in ICU significantly improved culture indices, leukocyte, white blood cell count, chest X-ray, chest secretion, CPIS score and saccharification (SpO2) on the third and seventh days after treatment compared to the first day.

**Conclusion:**

Considering the positive effect of adding inhaled colistin to the treatment of patients admitted to ICU with pulmonary infections caused by ventilator with multi-drug resistant Acinetobacter, the use of combination drug therapy is recommended.

## INTRODUCTION

A large number of studies have shown that Ventilator-Associated Pneumonia (VAP) is the most common nosocomial infection and one of the most frequent complications among patients admitted to hospitals and particularly for Intensive Care Unit (ICU) patients (1–5). As a result of recent researches, intubation with mechanical ventilation raises the risk of pneumonia by 6 to 20 fold among patients and is associated with 20–40 percent crude mortality rates (6,7). Pneumonia caused by VAP is the most common fatal infection which demands for prescription of parenteral antibiotics (7,8). About 20% of hospital-acquired pneumonia is due to using mechanical ventilation (9). The rate of mortality for nosocomial pneumonia ranges from 38 to 70%, and even higher rates for Multidrug Resistant Gram-Negative (MDR-GN) organisms (10). Recent studies indicate that the incidence of lower respiratory tract infection due to MDR pathogens and gram-negative bacilli such as *Acinetobacter baumannii* has been increasing (6,7). Therefore, according to the 2005 American Thoracic Society (ATS) and Infectious Diseases Society of America (IDSA) guidelines for management of VAP, improvement in patient outcome emphasizes the importance of appropriate antibiotic therapy based on patient risk factors for infection due to MDR pathogens (6).

Because of potent bactericidal action of colistin, low incidence of resistances, and excellent activity against gram-negative bacilli, even MDR strains, colistin was used as a polymycin antibiotic agent in the 1960s and 1980s (11). Colistin does not increase cross-resistance of gram-negative bacilli and its’ mechanism of action makes it less susceptible to bacterial resistance mechanisms (12,13). Over 20 years, inhaled colistin has been used successfully to prevent and cure pulmonary infections in cystic fibrosis patients (14). There are only few studies available assessing the effectiveness of inhaled colistin for the treatment of nosocomial pneumonia and particularly VAP (15,16).

Inhaled colistin has been used for both early and chronic infection in Europe and in the United States. The evidence from large controlled trials would support its use (17–23). But there are limited and conflicting data on the efficacy and adverse effects regarding inhaled colistin in the treatment of VAP in patients (24–26). This study compared the efficacy and safety rate of administering inhalation and intravenous colistin versus only intravenous colistin in patients with MDR-GN VAP.

## MATERIALS AND METHODS

Base on inclusion and exclusion criteria, the study population consisted of 114 VAP patients admitted in ICU of Hazrat-e Rasool General Hospital, Tehran, Iran (from March 2016 to April 2017). Ethical approval for the study was granted from the hospital’s review board. The clinical trial of the present study was registered in the Iranian Registry of Clinical Trials (IRCT) with the code number of (IRCT2015071323184N1).

### Inclusion criteria

- Age ranging: over 16 years
- Multi-drug resistant to MDR Acinetobacter
- Without sensitivity to polymyxins
- Positive culture (Colony count: Bronchoalveolar Lavage-BAL 10^4^ or Mini BAL 10^5^)

### Exclusion criteria

- Patient death
- Incidence of sensitivity to polymyxins
- Reduced Glomerular Filtration Rate (GFR) <0.25
- Incidence of bronchospasm evidences in treatment group

### Study design

In this blinded clinical trial, patients diagnosed with VAP entered the study. Patients who fulfilled all inclusion/exclusion criteria were randomly assigned into two groups. Randomization schedule was generated by using a random numbers table. In treatment group, patients received 150 mg colistin administrated intravenously in addition to 1,000,000 units inhaled colistin for one week every eight hours. The control group only received 300 mg of colistin every eight hours intravenously for one week (routine treatment).

On the first, third and seventh days after intervention, the patients were sampled and followed in terms of clinical and laboratory findings such as fever (>38°C), number of leukocytosis, plasma creatinine, plasma urea, SpO_2_, FiO_2_, blood pressure, heart rate, body temperature, and number of breaths per minute. Also on the seventh day, re-cultivation was carried out and in terms of changes in the nature of the patient’s Clinical Pulmonary Infection Score (CPIS) score was checked by the doctor using the same procedure.

### CPIS test Characteristic

The CPIS was calculated as an appropriate tool to facilitate the diagnosis of VAP (27). Luna et al. at baseline calculated and modified the CPIS by using the five variables (28). By adding the progression of the infiltrate and culture results of the BAL, CPIS at 72 hours was calculated based on seven variables (29). Papazian et al. used the CPIS for 38 patients who died after more than 72 hours of mechanical ventilation, of which 18 patients had histological evidence of pneumonia (30). The CPIS test of tissue samples is used as a gold standard for diagnosis due to the strength of its analysis. The study findings showed that for the presence of VAP, at the threshold of 6 points, CPIS had a high sensitivity, specificity and overall accuracy (30). Another study conducted by Fabregas et al., validated the CPIS by the presence of both histological and positive microbiological evidence of pneumonia in patients receiving mechanical ventilation. In Fabregas study, 25 patients died while receiving mechanical ventilation for more than 72 hours and the sputum samples were obtained immediately after death and before discontinuation of mechanical ventilation. The rate of sensitivity, specificity and overall accuracy of the presence of VAP in CPIS were high and similar to the Papazian study (30,31).

### Statistical analysis

Variables were evaluated for normality of distribution by the use of the Kolmogorov–Smirnov test. Quantitative variables are expressed as mean values ± SDs. Categorical variables were compared using Wilcoxon (to compare changes during treatment) or Mann-Whitney U test (to compare two groups), whichever was appropriate. Continuous variables were compared using paired sample t test (to compare changes during treatment) or student’s ***t*** test (to compare two groups). Statistical significance was defined as P value<0.05.

## RESULTS

One hundred and fourteen patients with MDR-GN pneumonia were studied: 57 patients received colistin intravenously in addition to inhaled colistin and 57 patients received only intravenous colistin. The average daily mortality rates for the first 7 days of the post-treatment period were less in treatment group than in the control (14 vs. 15.8%; P value=0.7). The side effect incidence rates (kidney diseases; urea level rise over 50%) were higher in control group on 3^rd^ day (5.4 vs. 3.6%; P value=0.6) and 7^th^ day (7.2 vs. 5.4%; P value=0.6). It should also be mentioned that one patient in the treatment group died after treatment.

Patient’s demographic information is listed in table 1. The two groups were similar in demographic characteristics. In terms of baseline characteristics, there were not significant differences between the two groups (P value > 0.05), except in case of Mean Arterial Pressure (MAP) and leukocyte count on 3^rd^ day in which there was a significant difference between the two groups (P value<0.05) (Table 2). On other hand, significant changes were observed during the treatment days in clinical and laboratory characteristics (Paired sample t test; P value<0.05).

**Table 1.** Demographic data of participants

**Parameter**	**Treatment group**	**Control group**	**P value**
**Age (y) ± SD**	49.19 ± 21.6	47.3 ± 18.3	0.61
**Male gender, n**	40 (70.2%)	40 (70.2%)	1
**Weight (kg) ± SD**	72.38 ± 10.67	73.33 ± 8.9	0.6

P value: comparison of demographic parameters communication between two groups by t test or Mann-Whitney U

**Table 2.** Comparison of clinical and laboratory characteristics in treatment and control groups (T test) on 1^st^, 3^rd^ and 7^th^ day after treatment

**Baseline characteristics**	**Days**	**Treatment group**	**Control group**	**P value**
MAP (Mean Arterial Pressure) ± SD	1^st^ day	97.2±21	92.6±17.7	0.2
	3^rd^ day	92.9±16	100.4±15.4	0.01
	7^th^ day	93±14.3	89.2±13	0.1
Heart rate (beat/min) ± SD	1^st^ day	99.7±26.2	107±26.7	0.1
	3^rd^ day	89.9±20.7	96.7±23.5	0.1
	7^th^ day	86.8±20.4	91.3±20.8	0.2
Respiratory Rate (beat/min) ± SD	1^st^ day	25.4±4	26±4.4	0.4
	3^rd^ day	19.9±3.2	21.3±4.3	0.07
	7^th^ day	18.1±3.9	18.3±4.5	0.7
Temperature (°C) ± SD	1^st^ day	38.5±1.1	38.5±1	0.7
	3^rd^ day	37.7±0.7	37.9±0.7	0.2
	7^th^ day	37.3±0.6	37.3±0.6	0.8
Leukocyte count (per mm^3^) ± SD	1^st^ day	14145.6±4759	15687.7±3899	0.06
	3^rd^ day	11303.5±4408	13635±4013	0.004
	7^th^ day	10269.6±3446	11064.9±3588	0.2
Plasma Creatinine ± SD	1^st^ day	1.2±0.5	1.2±0.6	0.9
	3^rd^ day	1.2±0.6	1.1±0.8	0.8
	7^th^ day	1.2±0.7	1.2±0.8	0.9
Plasma urea± SD	1^st^ day	30.2±10.8	26.5±11.2	0.07
	3^rd^ day	29.1±11.3	25.9±11.4	0.1
	7^th^ day	29.4±13.3	25.8±12.7	0.1
Arterial blood pH± SD	1^st^ day	7.4±1	7.4±1	0.8
	3^rd^ day	7.3±0	7.4±0	0.6
	7^th^ day	7.3±0	7.3±0	0.8
Pressure of oxygen in arterial± SD	1^st^ day	84.7±9.9	83.1±7.8	0.3
	3^rd^ day	87.9±9.9	86.5±7.3	0.3
	7^th^ day	89.4±11.8	89.3±8.5	0.9
Oxygen saturation (SpO_2_) ± SD	1^st^ day	92.1±6.3	89.7±6.4	0.052
	3^rd^ day	94.5±6	92.7±6.3	0.1
	7^th^ day	95.1±7.4	94.7±6.9	0.8
Fraction of inspired oxygen (*FiO*_2_) ± SD	1^st^ day	56±16.4	58.7±15.6	0.3
	3^rd^ day	51.7±17.7	54.9±16.5	0.3
	7^th^ day	49.7±17.5	54.3±18	0.1

CPIS characteristics were assessed and the results demonstrated in table 3. The Mann-Whitney U test showed that there was a significant difference only in the position of CXR, secretion and sputum culture of patients between the two groups on 3^rd^ and 7^th^ days (P value < 0.05). Also the CPIS in treatment group had a better score as compared with the control group on 3^rd^ and 7^th^ days (t test; P value = 0.0001).

**Table 3.** Comparison of Clinical Pulmonary Infection Score (CPIS) in treatment and control groups (T test) on 1^st^, 3^rd^ and 7^th^ day after treatment

**CPIS characteristics**	**Category**	**Treatment group**			**Control group**		

		**1^st^day**	**3^rd^day**	**7^th^day**	**1^st^day**	**3^rd^day**	**7^th^day**
**Temperature (°C)**	Normal	6(10.5%)	43(75.4%)	50(87.7%)	8(14%)	32(56.1%)	49(86%)
	low-grade feve***r***	27(47.4%)	8(14%)	2(3.5%)	23(40.4%)	20(35.1%)	4(7%)
	high fever	24(42.1%)	6(10.5%)	4(7%)	26(45.6%)	5(8.8%)	4(7%)
**White blood cells**	Normal	9(15.8%)	20(35.1%)	37(64.9%)	2(3.5%)	17(29.8%)	35(61.4%)
	<4 >11	47(82.5%)	37(64.9%)	19(33.3%)	55(96.5%)	40(70.2%)	22(38.6%)
	Band>500	1(1.8%)	-	-	-	-	-
**PaO_2_/FiO_2_**	Normal	49(86%)	51(89.5%)	51(89.5%)	48(84.2%)	49(86%)	49(86%)
	No ARDS	8(14%)	6(10.5%)	5(8.8%)	9(15.8%)	8(14%)	8(14%)
**CXR**	Normal	2(3.5%)	13(22.8%)	30(52.6%)	8(14%)	10(17.5%)	12(21.1%)
	Diffused/patchy	22(38.6%)	19(33.3%)	11(19.3%)	8(14%)	8(14%)	7(12.3%)
	Localize	33(57.9%)	25(43.9%)	15(26.3%)	41(71.9%)	39(68.4%)	38(66.7%)
**Secretion**	Normal	-	4(7%)	27(47.4%)	-	-	2(3.5%)
	Non purulent	1(1.8%)	30(52.6%)	24(42.1%)	-	3(5.3%)	36(63.2%)
	purulent	56(98.2%)	23(40.4%)	5(8.8%)	57(100%)	54(94.7%)	19(33.3%)
**Culture**	Normal	-	10(17.5%)	44(77.2%)	-	-	30(52.6%)
	Positive	57(100%)	47(82.5%)	12(21.1%)	57(100%)	57(100%)	27(47.4%)
**CPIS score**		7.9±1.3	5.4±2.1	2.5±2.7	8.1±1.4	6.86±1.6	4.5±2.4

## DISCUSSION

This study demonstrated that patients with MDR-GN pneumonia treated with both inhaled colistin and IV colistin compared to patients who received IV colistin had a more favorable outcome. The results of the study showed that during 7 days after treatment, inhaled colistin was well tolerated and the speed of recovery of patients in treatment group was higher than the control group which was in the same line with Korbila et al. (26). Also the mortality rate in both groups was approximately equal. It should be mentioned that the studied patients in both groups were matched in terms of age, gender and weight in the two groups.

Colistin or polymyxin E identified as a polymyxin antibiotic is produced by certain strains of Bacillus polymyxa var in 1949 (32). Because of the inherent resistant of Gram-positive bacteria, *Burkholderia cepacia*, *Serratia marcescens*, *Moraxella catarrhalis*, Proteus spp., Providencia spp., and *Morganella morganii* to the treatment with colistin, the limited range of colistin usage is only for treating the infections with *Pseudomonas aeruginosa* (*P. aeruginosa*) and *A. baumannii* (32). This polypeptide antibiotic is effective against most Gram-negative bacilli (33). Due to the absence of effective alternative cure, IV colistin has been used in patients with VAP caused by MDR-GN pathogens as salvage treatment (33,34).

Results of studies on the effect of direct delivery of antibiotics such as colistin were clinically beneficial to patients by increasing topical drug levels (35–37). Due to the increase of morbidity and mortality rate in patients with MDR-GN infections, it is important to determine if inhaled colistin had better healing results (25). Aside from the intravenous administration of colistin, it can be used as inhaled colistin whose effectiveness has been proven (18). Some studies reported that combination therapy (inhaled colistin as adjunctive therapy to IV colistin therapy) had high clinical and microbiological responses to VAP (38–41), which was similar to the results of this study. By contrast, in some other studies no additional benefit was observed (25,42).

The result of the present study and Korbila et al. could not show any significant difference in mortality rate between the two groups (26). So it seems that this therapeutic approach does not have definite impact on the mortality rate. But adding inhaled colistin to IV could significantly improve negativization of respiratory cultures.

The most important side effects of colistin therapy are nephrotoxicity and neurotoxicity which is more prevalent in patients with abnormal creatinine levels. Even during 1970s and 1980s, colistin was forbidden because of these difficulties (43). Several published studies confirmed the safety and efficacy of colistin caused by MDR-GN bacteria (44,45); but renal dysfunction is the most frequent reported adverse effect in the critically ill patients in the ICU setting (46). In other studies, the incidences of nephrotoxicity were reported as the main limiting factor for colistin usage in adults (24,38, 47). In the current study a total of 5 patients in treatment group and 7 patients in control group experienced nephrotoxicity after treatment. Although the incidence of nephrotoxicity was higher in control group but it was not statistically different.

The current study had some limitations that should be mentioned. It was not possible to control the particle size of real inhaled colistin which was administered via conventional nebulizers and as a result the exact amount of drug delivered to the lungs could not be exactly determined (48,49). We also should note that this was a retrospective study and only single-center site. This comparative evidence is only available to make a decision between IV antibiotics and inhaled colistin treatment to determine usefulness of inhaled colistin for VAP at that time.

## CONCLUSION

The results showed that receiving 150 mg colistin in addition to 1,000,000 units of inhaled colistin (inhaled-intravenous colistin therapy) had a better outcome compared to IV colistin alone in the treatment of VAP caused by MDR-GN Acinetobacter. On other hand, super-infection with inherently colistin-resistant pathogens and development of colistin option for multidrug resistant *P. aeruginosa* as an important issue should be assessed in future studies.

The patient’s death, patient’s withdrawal from the ICU and follow-up of patient’s problems were the main reasons for not checking the 30-day outcome, which were the limitations of this study.
